# Towards a Greater Understanding of the Role of the Environment: A Systematic Review of Qualitative MZ Twin Differences Studies

**DOI:** 10.1007/s10519-025-10217-1

**Published:** 2025-02-23

**Authors:** Filip Marzecki, Kennath Widanaralalage, Nandini Bhandoh, Tom A. McAdams, Yasmin I. Ahmadzadeh, Helena M. S. Zavos

**Affiliations:** 1 Department of Psychology, Institute of Psychiatry, Psychology & Neuroscience, King’s College London, Addison House M.02, Guy’s Campus Great Maze Pond, London, SE1 1UL UK; 2 Social, Genetic & Developmental Psychiatry Centre, Institute of Psychiatry, Psychology & Neuroscience, King’s College London, London, UK

**Keywords:** MZ Twins, Qualitative, Environment

## Abstract

The environment is an important influence in the development of human behaviours and health outcomes. However, one of the most consistent findings from behavioural genetic studies is that most environmental influences are not shared between members of the same family. A compelling way of investigating these non-shared environmental influences is by using a monozygotic (MZ) twin differences design. Quantitative MZ differences studies have uncovered systematic non-shared environmental factors, i.e., those acting according to a general pattern in a population, for many traits, but may be omitting idiosyncratic or distinctive factors and mechanisms. Qualitative MZ differences design provides an alternative. In this study design, identical twins discordant on an outcome are interviewed in depth about the origins and context of their discordance, providing an insight into distinctive lived experiences. We conducted a systematic review examining the results and methodological features of studies using qualitative data collection and analyses to investigate differences in identical twins' experiences and outcomes. We applied a narrative synthesis. We identified seven studies, covering a range of phenotypes (e.g., anxiety or smoking) and participants (from children to older adults), which found a wide range of themes related to twins’ discordance. A major theme arising from the narrative synthesis was the role of personality and individual traits, e.g., confidence or sexual orientation, in explaining MZ twins’ discordant experiences and outcomes. Non-shared environmental factors are at least partly idiosyncratic and are therefore suitable for exploration with a qualitative research design, ideally in parallel with quantitative twin research in mixed-method research projects or programmes.

**Supplementary Information:**

The online version contains supplementary material available at 10.1007/s10519-025-10217-1.

## Introduction

For most behavioural traits the environment accounts for more than half of the variance, and this is largely driven by unique environmental experiences (Turkheimer 2000; Plomin 2011). These are often referred to as non-shared environmental influences (NSE), i.e., factors that make members of the same family different to one another, which include different parental treatment and experiences outside of the family, such as friendships. The influence of the NSE can be estimated from family study designs, such as twin studies. However, the systematic approach of quantitative twin studies might not capture distinctive and subjective experiences that explain some twins’ discordance and the context of individual differences between identical twins.

The quasi-experimental MZ differences design is a genetically informed design well suited to studying NSE influences and can help elucidate the possible causal role of specific environments on behavioral and health-related traits and outcomes. Monozygotic (MZ) twins share all their segregating genes and some family-based environmental exposures (e.g., childhood SES, parental divorce), therefore most differences that occur in their behaviour or characteristics can be attributed to the NSE. With binary exposures and outcomes, the MZ differences design compares outcomes between the exposed twin and the nonexposed twin within an identical twin pair (Carlin et al. 2005; McAdams et al. 2021). With continuous exposures and outcomes, the difference score in exposure between twins is first estimated and then tested for association with difference in outcomes (McAdams et al. 2021). The within-pair relationship between exposure and outcome can be estimated through a range of statistical frameworks, such as generalised estimating equations or structural equation modeling (Carlin et al. 2005). Due to its quasi-experimental nature, the MZ differences design has had great utility for health-related and psychological research. For example, it has demonstrated a causal link between smoking and lung cancer (Hjelmborg et al. 2017), bullying victimisation and common mental health difficulties (Singham et al. 2017), or childhood maltreatment and mental health difficulties (Baldwin et al. 2023).

However, the NSE of meaningful influence on behavioural traits may be unsystematic, i.e., not acting according to a general pattern in a population—an argument first proposed by Plomin and Daniels (1987). They referred to this as the “Gloomy Prospect”, because of the relative difficulty of identifying salient environmental factors that may not be systematic in their effects across people. This claim was supported by a review of 43 studies, which found that objectively measured NSE factors accounted for on average < 2% of the variance in behavioural outcomes (Turkheimer and Waldron 2000). Quantitative research and statistics used for most behavioural research are nomothetic, meaning that they provide a comprehensive view of general patterns and universal principles relevant to a population. Quantitative methods are not able to capture the depth of NSE factors, including differences in interpretations, on discordance among identical twins. It may be that reducing the complexity of NSE to so-called objective and quantitative measures is one reason why it remains so difficult to answer the question of what the salient NSE factors are.

Qualitative research can add to research on environmental effects on behaviour as its idiographic nature means that it can focus on distinctiveness, context, and the subjective experience—all the things that quantitative twin studies are unlikely to highlight. Qualitative methods are aimed at understanding a participant’s social setting by learning about their experiences, perspectives, and life circumstances (Moriarity 2011). They help in the development of meaningful explanations by evaluating all data equally, even that brought up by a single participant (Sofaer 1999). Qualitative methods spotlight the participants’ experiences through open questions, e.g., “how and why were your working conditions stressful?”, rather than “were your working conditions stressful?”. The qualitative approach allows for an inductive exploration, unlike quantitative research, which limit the participants’ experiences to what is included on the questionnaires. This makes qualitative research relatively less guided by the researchers’ expectations and hypotheses.

In application to the field of behavioural genetics, qualitative methods may be useful for understanding NSE factors, explanations for environmental mechanisms and the context of individual differences. A qualitative MZ differences study uses qualitative forms of data collection and analysis, like semi-structured interviews and thematic analysis, instead of quantitative data collection and analysis, to examine differences in experiences of monozygotic twins. Here, qualitative data allows discordance among MZ twins to be investigated in greater depth, whilst controlling for genetics and shared environment. For example, a twin pair interviewed in one qualitative study of MZ twins discordant for depression were found to be discordant in exposure to a single traumatic injury, but the in-depth interview shone light on the unique process through which the traumatic event influenced the development of depression (Kendler & Halberstadt 2013). Namely, the affected twin had a significant accident in childhood which impaired their cognitive function and in turn their access to higher education and a stable, well-paid career. The twin who had experienced the injury had subsequently struggled with employment and finances, which they perceived as a significant contributing factor to their depressive episodes. Without the depth and richness of information granted by the qualitative approach, it could be concluded that the accident was a key cause for this individual’s depression. However, as one learns from the autobiographical interview, the social disadvantage faced due to their disability was an important factor for their mental health difficulties. This highlights that qualitative methods can help in understanding complicated processes behind human behaviour.

Despite being a promising avenue for exploring the NSE and individual differences, qualitative methods have rarely been applied to study discordance in identical twins. To our knowledge, there are a few studies that explicitly apply the qualitative MZ differences research design, alongside some published qualitative case studies of discordant MZ twins. Such case studies appear to focus on rare outcomes. For example, one publication reports on three twin pairs, using qualitative methods, who were discordant for schizophrenia or schizoaffective disorder, whilst being concordant for obsessive–compulsive disorder (Lewis et al. 1991). Although the authors report on three twin pairs, they describe each pair with no analysis of qualitative data that would lead to a synthesis of findings across the pairs. The descriptive nature of case studies means that they do not apply qualitative analysis methods to synthesise data for overall conclusions. It is imperative that research not only gathers but also analyses non-numerical data to be considered qualitative research. Additionally, some studies use qualitative methods in family research and even with families of twins, for example to investigate the mothers’ experiences (Caspi et al. 2004). This is another avenue for applying qualitative methods in family research. However, in this review, we focus exclusively on the experiences of twins, considering studies that apply qualitative research methods to explore the differences in their behavioural and health-related traits and outcomes.

To foster the development of the qualitative MZ differences design, we have conducted a systematic review of the current literature. The aim of this review was to examine the insight gained by the qualitative MZ differences studies so far, as well as their methodological features and quality. With regards to the second aim, we focused on the following features important for rigorous qualitative research: recruitment, data collection methods (e.g., interview, focus group), epistemological approach if discussed (e.g., positivism, interpretivism), data analysis method and methodology if discussed, and sample characteristics (e.g., size, average age).

## Methods

This review was preregistered on Prospero—CRD42023471737 (available from: https://www.crd.york.ac.uk/prospero/display_record.php?ID=CRD42023471737).

### Search Strategy

Searches were carried out in two databases (APA PsycINFO and PubMed) in November 2023. Manual searches were also conducted by searching reference lists of included studies and checking for published papers associated with any relevant conference abstracts. The search was restricted to articles in English, with any publication date, and unpublished studies were not sought. The searches were re-run in November 2024 and no new articles were identified.

Three core concepts were used to construct the search terms: (1) identical twins; (2) qualitative research; (3) discordance. Multiple terms were used within each of these concepts, and the full search strategy is provided in the supplement (Appendix 1). The terms within a concept were combined with Boolean operator OR, and then term clusters were combined with Boolean operator AND. To capture mixed method publications, we did not apply a methodological filter for qualitative studies.

### Study Selection

The pre-registered review protocol on Prospero outlines the eligibility criteria that were set for inclusion and exclusion of the study. The review was focused on participants who are identical twins reared together, open to any condition, characteristic or exposure that identical twins participating in a study were discordant on. For example, it could be but was not limited to mental health (e.g., depression, anxiety), neurodevelopmental conditions (e.g., autism), behaviours (e.g., substance use), or physical health (e.g., Type 1 diabetes). The review was focused on novel results from qualitative analyses conducted on data self-reported by MZ twins.

**Fig. 1:**
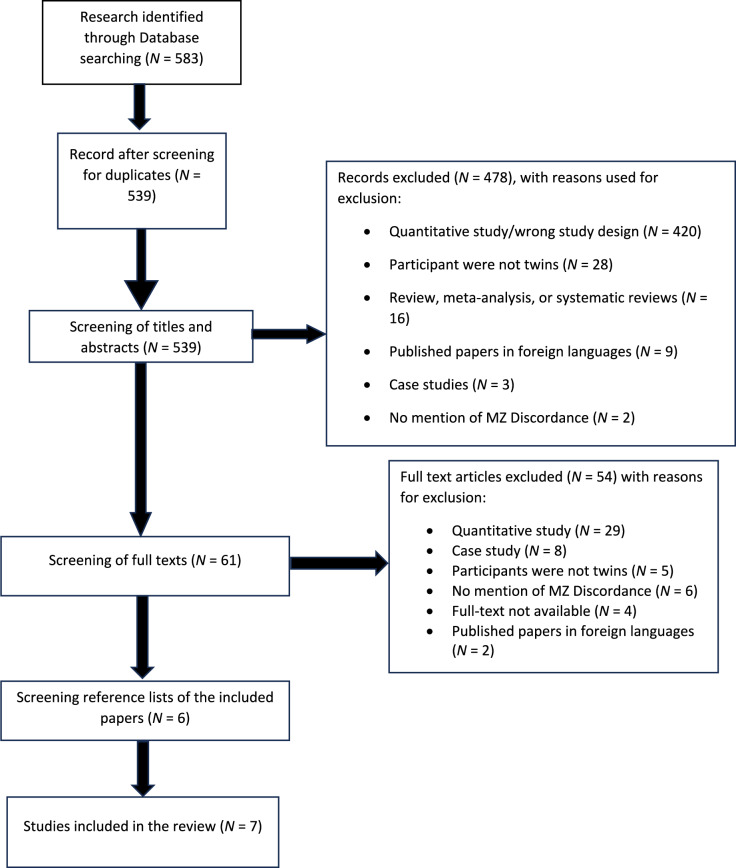
PRISMA flowchart of the screening process

EndNote, the reference manager software, was used to compile citations, titles, and abstracts from the databases search, removing any duplicates, thus creating a comprehensive database. Subsequently, the research team used Rayyan Software to assess the titles, abstracts, and full texts stored in the database. In the first step, eligibility criteria were applied by one reviewer, FM, to screen all the titles and abstracts. The second reviewer, HZ, independently double-screened at least 20% of the titles and abstracts, blinded to the decisions by FM. During this step, exclusion criteria were applied sequentially in the following order: not published in English language; review, meta-analysis, or systematic review; participants not twins; twins reared apart; wrong study design, i.e., an exclusively quantitative study, or not involving both qualitative data collection method and qualitative analysis method; no mention of MZ discordance. In the second step, full texts of the retained papers were screened by FM, and HZ independently double-screened at least 20%, to include or exclude in the review. In this step, exclusion criteria were applied in a random order for which feature was first noticed by the reviewer. The PRISMA diagram (Fig. 1) presents the number of primary records and the count of records meeting the inclusion criteria after evaluating titles/abstracts and full texts. In instances of disagreement in each of the stages, resolutions were reached through discussions between the reviewers (FM and HZ).

### Data Extraction

Data extraction was conducted by FM using a data extraction form in Microsoft Excel, checked by reviewers HZ and KW for accuracy and completeness. Data items to be included in the form were: publication details (author, title, year of publication), research question, discordant trait, the main findings, recruitment strategy (e.g., birth cohort study, or general population), sample characteristics (e.g., size, average age), data collection methods (e.g., interview, focus group), whether study was qualitative or mixed methods, epistemological approach if discussed (e.g., positivism, interpretivism), and qualitative methodology if discussed (e.g., Grounded Theory).

### Quality Assessment

The Critical Appraisal Skills Programme (CASP 2013) provides a quality assessment checklist for qualitative research, and this checklist was applied to this review. The checklist provides questions, such as “Was the data collected in a way that addressed the research issue?”, which can be answered with “Yes”, “Can’t Tell”, and “No”. The checklist allows for a free text comment to each of the questions/criteria and these comments were used to narratively appraise the studies in the results.

### Data Synthesis

Due to the qualitative nature of this review and the fact that we were reviewing studies addressing a wide range of research questions, we synthesised the data narratively. Tables with a summary of findings are provided in the results. A further narrative appraisal of the quality of the studies is provided in-text, as per our quality assessment (CASP 2013), alongside an outline of the results provided in one of the tables (methodological features).

## Results

The search of the databases identified 539 papers after removing duplicates. After screening the titles and abstracts, 61 records were retained for full-text screening. After the full-text screening, 6 papers were included in the review and one further relevant paper was identified through manual searches, in the reference list of one of the 6 selected articles (see Fig. 1 for the PRISMA Flow Chart). All included papers were published in the last 20 years and no paper studied the same discordant trait or experience. The studied traits or experiences included mental health conditions (e.g., Major Depressive Disorders), behaviours (e.g., instrument playing), and environmental experiences (i.e., peer relationships). The characteristics of the included studies can be found in Table 1.

**Table 1:** Summary of qualitative MZ differences studies

Authors and Date	Title of the article	Main aim	Discordant trait	Main findings
Asbury et al. (2016)	Nonshared Environmental Influences on Academic Achievement at Age 16: A Qualitative Hypothesis-Generating Monozygotic-Twin Differences Study	To generate hypotheses about what non-shared environmental factors influence academic achievement at age 16	Academic achievement at age 16	Discordance in academic achievement at age 16 (i.e., UK GCSE level) appeared related to discordance in school experience (e.g., ability grouping, perceived teacher quality, and perceived teacher–pupil relationships) or in behavioural traits (differential effort, ability, interest, and personality)
Asbury et al. (2017)	Do MZ twins have discordant experiences of friendship? A qualitative hypothesis-generating MZ twin differences study	To understand whether MZ twins have different experiences of peer relationships, and how they percieve peer relationship discordance, i.e., whether they perceive it as causal, consequential (selection hypothesis) or correlated to discordant behaviour	Peer relationships	Peer relationships discordance fell into six categories (e.g., peer victimisation). The NSE source of the discordance was perceived to be due to chance, vulnerability of one twin, or discordant behaviour. Discordant peer relationships resulted in discordant self-confidence, future plans, social isolation, mental health, and interests
Asbury et al. (2006)	The Use of Discordant MZ Twins to Generate Hypotheses regarding Non-shared Environmental Influence on Anxiety in Middle Childhood	To generate new hypotheses regarding specific non-shared environmental influences on childhood anxiety by asking anxiety-discordant MZ twins and their mothers to describe the children’s differential experiences	Anxiety	Sources of discordance in anxiety in middle childhood were negative school experiences, comparisons within the twin relationship, and illnesses and accidents, traumatic neonatal events (breathing problems, separation from mother), parent–child relationships, peer rejection
Eriksson et al. (2017)	Similar but different: Interviewing monozygotic twins discordant for musical practice	To investigate environmental influences on music practice in pairs of MZ twins discordant for piano playing	Keyboard instrument playing	Sources of difference were concluded to vary by individual. Different relations to music rather than systematic environmental differences appeared to be the antecedent factors for discordance in playing keyboard instruments. Playing twins had more interest and motivation to engage in music, found it more important for everyday life and some connected music to their personal identity
Kendler and Halberstadt (2013)	The road not taken: life experiences in monozygotic twin pairs discordant for major depression	To understand how non-shared environmental experiences contribute to major depression	Major Depressive Disorder (MDD)	Themes identified as reasons for discordance in major depression were differences in romantic relationships, differences in being planful, as well as occupational stressors (e.g., lack of a stable career) and single traumatic events. The path from romantic relationships to depression was unclear, as it could be bidirectional, and choice of partners could be influenced by depression-related personality differences
McLeod et al. (2008)	How do friends influence smoking uptake? Findings from qualitative interviews with identical twins	To understand how friends influence smoking uptake	Smoking	Discordance in friendship played an important role in discordance in starting and continuing to smoke. Smoking was identified as a mechanism for joining and staying connected with a group who smoked. For non-smoking co-twins, the image of social groups who smoked was at odds with their self-image, and this contributed to their non-smoker status. Overall, it was concluded that images of smoking as well as non-smoking are used to construct social identities of individuals and groups
Pearsall-Jones et al. (2011)	Monozygotic twins concordant and discordant for DCD: two sides to the story	To explore perceptions of family dynamics, including relationship with co-twins, in families of MZ twins in which either one or both had a movement disorder	Developmental Coordination Disorder (DCD)	No differences in the perception of the twin relationship were found in pairs discordant for DCD. Most twins felt that they could talk to their co-twin about anything without fear of judgement and that the co-twin provided a buffer again anxiety-provoking situation (e.g., changing schools)

Four studies had the aim of investigating NSE influences on a discordant outcome. For example, Eriksson et al. (2017) sought to investigate environmental experiences that were related to discordant status in keyboard instrument playing. Another study sought to investigate how a particular environmental experience (friendships) influenced a twin pair’s discordance in smoking behaviour (McLeod et al. 2008). One study had the aims of investigating NSE influences on peer relationships, treating these as an outcome (i.e., what NSEs can lead to discordance in peer relationships) as well as treating discordance in peer relationships as a NSE that can influence further discordant outcomes (Asbury et al. 2017). Finally, one study focused on discordance in developmental coordination disorder as a factor potentially influencing the perception of family dynamics (Pearsall-Jones et al. 2011).

### Methodological Features of the Studies

Methodological features of these studies are summarised in detail in Table 2. All the studies recruited participants from existing twin cohort studies or registries. Sample sizes were generally modest as typical for qualitative research (7–112 discordant MZ pairs) with the majority including less than 20 pairs. The studies involved participants from a range of ages—including children (age 8 in Pearsall-Jones et al. 2011, and Asbury et al. 2006) and older adults (age 65 in Kendler and Halberstadt 2013). None of the studies reported on the ethnic or racial identities of the participants. All studies were conducted in High Income Countries: England, Wales, Sweden, US, or Australia. Nearly all the studies collected the data through semi-structured interviews. Two studies developed bespoke interview guides for each participant, based on within-twin-pair differences identified in a pre-interview questionnaire (Asbury et al. 2016, 2017). One study organised the interview into five sections based on pre-decided themes, some of which were based on prior literature on the topic (Eriksson et al. 2017). The remaining articles lacked detail on how the researchers developed the semi-structured interview questions. One study (Kendler and Halberstadt 2013) conducted autobiographical interviews. Most of the studies ascertained discordance through quantitative data collection, such as surveys or diagnostic interviews. In one study, discordance in peer relationship was designated if spontaneously mentioned by parents and/or twins in a questionnaire and/or telephone interview about non-shared environment (Asbury et al. 2006).

**Table 2:** Methodological features of qualitative MZ differences studies

Author and date	Participant recruitment strategy	Country	Sample size	Age of twins	Data collection method	How was discordance ascertained?	Epistemological stance	Qualitative analysis method
Asbury et al. (2016)	Recruited from participants of Twins Early Development Study (TEDS), a longitudnal study of twins born between 1994 and 1996	England and Wales	56 MZ pairs (sex unreported)	Average 17 years old (range 16—18)	Semi-structured telephone interviews	Pairs were deemed discordant if their national school assessments (GCSE scores) were at least two grades apart in at least one subject	Inductive	The framework approach (Ritchie and Spencer 2002) was applied, which involved synthesising data through five stages: familiarization, identifying conceptual themes, indexing, charting, and mapping
Asbury et al. (2017)	Recruited from participants of Twins Early Development Study (TEDS), a longitudnal study of twins born between 1994 and 1996	England and Wales	112 MZ pairs (sex unreported)	Average 17 years old	Semi-structured interview	Discordance was spontaneously mentioned by parent and twins in questionnaires and/or telephone interviews	Inductive	The framework approach (Ritchie and Spencer 2002) was applied, which involved synthesising data through five stages: familiarization, identifying conceptual themes, indexing, charting, and mapping
Asbury et al. (2006)	Recruited from participants of Twins Early Development Study (TEDS), a longitudnal study of twins born between 1994 and 1996	England and Wales	19 MZ pairs (15 female)	8 and 9 years old	Semi-structured interview with mother and twins (only mothers’ interview data was analysed)	Teachers rated twins' anxiety and those with discordance 2 standard deviations or more above the mean discordance for the sample were selected. Furthermore, the discordance was validated through restricting the sample to the pairs who were also rated one standard deviation or more above the mean discordance rated by the parents as well as the pairs in which the same twin was rated as more anxious by the parent and the teacher	Inductive	No direct mention, but the authors identify themes through their analysis
Eriksson et al. (2017)	Recruited from a sample of 11,543 twins from the STAGE Cohort of Swedish Twin Registry, born between 1959 and 1985 who participated in a web survey in Autumn 2012	Sweden	10 MZ pairs (6 female)	Range 31–47 years old	Semi-structured interview	Discordance on a web-survey for self-reports of playing a keyboard instrument (piano, organ, or keyboard—yes or no), and within-pair difference in total hours of music practice > 1,000 h	Deductive—semi-structured interview questions rooted in prior literature and knowledge	No direct mention, but the authors develop categories and identify themes in their analysis
Kendler and Halberstadt (2013)	Recruited from participants of the Virginia Twin Study of Psychiatric and Substance Use Disorders	USA	14 MZ pairs (8 female)	Range 42–65 years old	Joint autobiographical interviews	As part of the Virginia Twin Study of Psychiatric and Substance Use Disorders, structured psychiatric interviews were conducted. To meet the discordance criteria, the affected twin had to have reported one or more lifetime episodes of MDD as defined by DSM-III-R criteria on at least two assessments, the unaffected twin had to have denied a lifetime history of episodes of MDD on all assessments and the age of the unaffected twin at last interview had to be at least 10 years older than the age at onset of MDD in their twin	Inductive	No direct mention, but the authors report autobiographies in major groupings and draw on themes across twin pairs
McLeod et al. (2008)*	Recruited from twins who participated in in a longitudinal study on smoking and drinking experiences during adolescence, previously recruited through The Australian National Health and Medical Research Council Twin Registry	Australia	14 MZ pairs (9 female)	Range 27–33 years old	Semi-structured telephone interviews	Data from the longitudinal surveys was used to identify identical twin pairs discordant for smoking. First, they defined twins as discordant if one twin had smoked in the week prior to the young adulthood survey and the other had not smoked in the past year. Second, they identified identified twin pairs that were discordant for smoking at the first survey (completed at 11–18 years old). They contacted these twins and asked about their smoking status, excluding pairs who were either not discordant for smoking at the time of the interview or have only been discordant for a few years	Inductive	No direct mention, but the authors cite a source for Grounded Theory analysis (Bryman and Burgess 2002)
Pearsall-Jones et al. (2011)	Recruited from the Australian Twin Registry	Australia	7 MZ pairs (4 female; additionally 2 MZ male pairs who were concordant)	Between 8 and 17 years old	Semi-structured interview	The twins' primary caregiver completed the Twin and Sibling Questionnaire, which incorporated the Developmental Coordination Disorder Questionnaire (DCD-Q) to ascertain discordant or concordant status	Inductive	Thematic analysis (Braun and Clarke 2006)

*McLeod et al. (2008) specified their methodology as Grounded Theory and the remaining studies did not mention methodology

The reviewed studies included limited detail on the analysis methods and methodologies (see Glossary in Appendix 2) used. With regards to the analysis methods (i.e., tools used to analyse data)), one study directly mentioned applying thematic analysis (Braun and Clarke 2006), another study mentioned applying Grounded Theory (Bryman and Burgess 2002) and two studies mentioned the framework approach (Ritchie and Spencer 2002). The remaining 3 studies did not mention a standardised technique. Analysis methods differ from methodology. Methodology refers to the theory of how research should be conducted to lead to valid knowledge (see Glossary in Appendix 2). Regarding this, only one study (McLeod et al. 2008) specified their methodology, which was Grounded Theory.

Finally, in assessing the epistemological stance taken by the researchers, the studies were categorised into deductive or inductive, based on whether they approached their research question with pre-specified hypotheses (deductive) or not (inductive). All the studies took an inductive approach, except for Eriksson et al. (2017).

### Insights About Differences Between Identical Twins

Here we conduct a synthesis of the study findings, while acknowledging that the conclusions concerning factors related to MZ twins’ discordance were specific to the life stage and the traits investigated in each study (for a summary of themes extracted in each study, see Supplementary Table 1). Studies that focused on broad NSE factors investigated twins discordant on academic achievement, anxiety, instrument playing, MDD, and peer relationships. One of the studies identified experiences of school (e.g., ability grouping) as well as personality and individual traits (e.g., effort and motivation for studying) to be NSE factors related to academic achievement (Asbury et al. 2016). In the study on anxiety in middle childhood, NSE explanations for discordance also included experiences of school, such as negative experiences of the anxious twin (e.g., bullying), as well as twin relationship (unfavourable comparison by the anxious twin). In fewer families, discordance in anxiety was explained by illnesses and accidents, such as appendicitis that led to continuous worry about pain, and traumatic neonatal events, such as the anxious twin born not breathing. Finally, different relationships with parents, e.g., the relatively smaller amount of affection between the mother and the anxious twin, and peer rejection were also suggested as explanations for some twin pairs’ anxiety discordance (Asbury et al. 2006).

In the study of peer relationships, the explanations varied by the type of peer relationships discordance. Concerning peer victimisation and rejection, chance as well as personality and individual traits, which enhanced one twin’s vulnerability, were highlighted (Asbury et al. 2017). For discordance in the number of friends, explanations of discordance included personality and individual traits (e.g., self-esteem), illnesses and accidents, as well as chance and romantic relationships. Similarly, discordance in friendship groups was explained by chance, personality and individual traits (e.g., interests), as well as parental encouragement. Finally, discordance in attitudes towards friendship was contextualised within differences in twins’ personality and individual traits (e.g., discordant efforts to socialise and different levels of confidence) and reactions to the twin relationship.

Personality once again came up as an explanation for identical twins’ differences in the study of musical practice discordance, where motivation and identity (i.e., as a musician or not a musician) played an important role in discordant outcomes. Furthermore, NSE influences on musical engagement included access to resources and social relationships, such as differences in musical interest in friendship groups and different relationships with the music teacher (Eriksson et al. 2017). The study on MDD identified personality related NSE influences (e.g., being planful), in addition to illnesses and accidents, social and romantic relationships, as well as reoccurring stressors (i.e., occupational stressors, such as lack of career stability; Kendler and Halberstadt 2013). As this study stood out in its lifelong NSE experiences approach (the only one in which participants were older adults), it may be that recurring stressors are an NSE factor that only becomes significant as one gets exposed to more experiences with age.

In the one study focused on specific explanations for discordance (in twin smoking behaviour), social relationships were an a priori focus, however, personality and individual traits, such as identity, came up as relevant for some participants. Apart from factors such as peer pressure, one’s social identity was described as influencing individuals’ smoking experimentation and consolidation, for instance, smoking co-twins described being attracted to the images related to the group who smoked and using smoking as a means for becoming part of that group (McLeod et al. 2008). On the other hand, non-smoking twins spoke about the images associated with the smokers’ groups as incongruent with their self-image and the image of their friendship group.

Finally, one study did not investigate explanations for discordance in a trait, but instead focused on experiences of MZ twins that may be resulting from discordance in having developmental coordination disorder (Pearsall-Jones et al. 2011). Their aim was to investigate how a specific factor relates to perceptions of family relationships. The results indicated no differences in perceived family dynamics in discordant twins. It should be noted that this study focused only on two specific factors (developmental coordination disorder and family relationships) and as such provides little insight into general NSE influences.

## Discussion

The aim of this systematic review was to synthesise the results of studies that used qualitative methods to examine differences in the experiences and outcomes of monozygotic twins (the qualitative MZ differences design), as well as to examine their methodological features and quality. This study design can spotlight complex processes behind twins’ discordant behaviour and health-related outcomes whilst excluding genetic and broad sociodemographic factors. It allows for an inductive exploration of non-shared environmental (NSE) factors and can spotlight distinctive lived experiences. We identified seven studies that used the qualitative MZ differences design. Their results highlighted a wide range of themes associated with discordant characteristics of a twin pair. For example, two studies investigating effects in childhood and adolescence (anxiety and academic achievement) found school experiences, such as ability grouping and teachers, to be NSE factors associated with discordant outcomes. Nearly all the studies drew conclusions about the role personality and individual traits play in MZ twins’ discordance, regardless of age and outcome. Some of these were motivation, confidence, individual identity and sexual orientation. Stressful environments (including illnesses and accidents, traumatic neonatal events, and occupational stressors) were also identified as NSE factors pertaining to differences in the number of friends, childhood anxiety, and depression. Interpersonal relationships, including social and romantic relationships as well as those with the co-twin, teachers and parents, arose as NSE factors in most of the studies. Finally, themes that came up in one study only were chance, choice and access to resources (more specifically to a piano).

The themes of MZ twin discordance within each study were multiple and not one of the studies drew a simple conclusion derived from a common pattern of reporting across participating twin pairs. This supports the view that many NSE factors are individual-specific, reinforcing to some extent the suggestions made in a seminal quantitative meta-analysis (Turkheimer and Waldron 2000). The qualitative MZ differences method has potential to explore this distinctiveness of the mechanisms by which environment may influence behavioural outcomes. Additionally, in our synthesis of the diverse experiences, studies and findings, we concluded that only some of the NSEs of meaningful influence concerns exogenous events, such as accidents and single traumatic events. We found that discordance in other individual differences, such as other traits, were often related to discordance in a studied outcome. For example, out of a pair of identical twins, a gay twin had a different experience of peer relationships, experiencing peer victimisation and rejection, unlike their straight co-twin. Finally, social, romantic and family relationships came up across different phenotypes, highlighting how much other people can shape our experiences.

There are limitations to qualitative MZ differences studies. The direction of effects is difficult to establish from qualitative data collection methods, and many of the differences between discordant twins must be viewed as correlational rather than causal. For example, even if twins described differences in being planful prior to one of them experiencing depression, a trait like being unable to stay organised and make plans can be a sign of mental health difficulties and thus far from the cause of them. The issue of the direction of effects could however be explored further by joining the forces of qualitative and quantitative twin studies in mixed-method research, whereby themes from a qualitative study could inform a quantitative study with more potential for causal inference, such as a longitudinal MZ differences study (McAdams et al. 2021). Another limitation is that only perceptible NSE factors can be explored this way, but it has been suggested that unsystematic NSE might also include internal factors, such intrinsic molecular stochasticity (Tikhodeyev and Shcherbakova 2019). Finally, our narrative approach to synthesising the findings has limitations, but due to heterogeneity of the studies and their limited number, a different synthesis method was not suitable. Once more qualitative MZ differences studies are conducted, such a review should be revisited with an a priori plan for a suitable meta-analysis, such as a meta-synthesis (Lachal et al. 2017) or a Meta thematic analysis (Sağlam et al. 2023). Additionally, we made the decision to exclude case studies from this review, because most case studies do not apply qualitative analysis methods. However, one recent case study applied collective autoethnography to investigate the phenomenology of eating disorders in identical twins (Elwyn et al. 2024). This highlights that there are other approaches to qualitative research with MZ twins beyond the analysis of interviews with several discordant MZ twin pairs. Future syntheses of qualitative MZ differences research could consider including these. Not least of all, there are methodological limitations pertaining specifically to some of the current literature, which we discuss in the following section.

### Methodological Characteristics of Qualitative MZ Differences Studies

In addition to synthesising the current literature, examining the quality and methodological features of qualitative MZ differences was the other aim of this review. Concerning methods of data collection, data was mostly collected via twin registries through inductive, semi-structured interviews with each twin separately. One study conducted joint autobiographical interviews. Future studies applying this design are advised to follow the same data collection methods. A limitation of most of the current literature is the lack of information on how the researchers developed their interview questions. Future researchers should be clear about how they did this, for example, by providing their interview guide in the supplementary materials. Autobiographical interviews could serve to make the approach more inductive, if this aligns with the research aims (for discussion of this method see Domecka et al. 2012). The interviews (semi-structured/autobiographical) should be conducted with each twin separately. Joint interviews can change the dynamic with the researcher and each twin’s responses might be influenced by those of their co-twin.

Concerning methods of analysis, only 3 studies directly stated their analysis method, which was either the framework approach (also called codebook thematic analysis; Ritchie and Spencer 2002) or (reflexive) thematic analysis (Braun and Clarke 2006). The application of thematic analysis in psychological sciences has been criticised on the basis that many researchers cite existing analysis methods, giving a general understanding without information regarding how this was uniquely applied to the present topic and study (Braun and Clarke 2023). The qualitative MZ differences studies conducted to this day mostly apply qualitative analysis within a positivist value framework (see Appendix 2) but they do not refer to this within their papers. In other words, their method is not explicitly linked to the theory behind it and the rationale for its use is not discussed. Future researchers should explain why their research aims are best addressed with a particular method of qualitative analysis. For example, they could state that reflexive thematic analysis was chosen as it allows for induction and the study’s aims are inductive.

Future researchers making use of the qualitative MZ differences study design should elaborate more on how they analysed the qualitative data. That is, explain what qualitative method they applied, how it worked specifically to address their aims, and why was it chosen. We recommend reflexive thematic analysis (for guidance on use, see Terry and Hayfield 2021) due to its theoretical flexibility, meaning that it can work with different theoretical approaches, such as positivism or constructivism (see Appendix 2), and can work for inductive or deductive aims (Clarke and Braun 2017). Reflexive thematic analysis is fitting for qualitative MZ differences research conducted as a means of supplementing quantitative twin research. As such, qualitative MZ differences studies may have inductive as well as deductive aims, depending on whether the aim is to generate hypotheses for quantitative twin research or to explain phenomena already explored in quantitative twin research in more depth.

### Reflexivity in Qualitative MZ Differences Research

For researchers trained primarily in quantitative methods, it is important to note the differences that characterise qualitative research. For one, qualitative research has different underpinnings to quantitative research, for example, it does not aim to identify universal trends, therefore needs to be interpreted with the researched population in mind. Qualitative research focuses on transferability (Treharne and Riggs 2015) as opposed to generalisability. It also does not aim to be unbiased but does aim to be transparent about the position of the participants and the researchers (Yardley 2017). This means that it is especially important to report on the characteristics of the sample (e.g., ethnicity and race, which were not reported by any of the included studies). Characteristics of the researchers doing the data collection and analysis should also be considered due to positionality and reflexivity (see Glossary in Appendix 2). Reflexivity refers to the examination of the researcher’s beliefs and judgments and how these may influence the research. This is important as the researcher is an active player in the conduction, analysis and interpretation of the research, therefore they cannot be separated from qualitative research (e.g., Levitt et al. 2017). Future researchers using qualitative MZ differences designs should seek to incorporate reflexivity into their analysis and interpretations of the research.

### Mixed-Method Twin Research

Qualitative MZ differences studies are a promising avenue as they have the potential to supplement the insight of quantitative twin studies. This research design is most likely to have utility in mixed-method research programmes, in conjunction with quantitative twin research, because if controlling for some common sources of variance (e.g., sociodemographic) is not the aim of the research, the researchers would likely conduct a qualitative study with a non-twin sample. In mixed-method research, the quantitative and qualitative processes should be integrated rather than conducted in parallel (Creswell and Clark 2011). Two mixed-method approaches that are relevant are Sequential Exploratory and Sequential Explanatory. The former is characterised by conducting a qualitative phase first that informs the quantitative phase, whereas the latter involves conducting a quantitative phase first and then exploring the findings of that phase in more depth in a qualitative phase (for more detail on mixed-methods and these two approaches, see Doyle et al. 2016). Both offer interesting opportunities for twin researchers—the sequential exploratory approach allows twin researchers to begin with generating hypotheses, which may be particularly useful in unexplored areas, whereas the sequential explanatory approach allows researchers to explore the complexity and the nature of NSE factors in more depth.

Finally, researchers interested in applying the qualitative MZ differences design should be ethically considerate. In this unique context, researchers have to be particularly mindful of disclosure between twins. In reporting on qualitative data from members of the same family, confidentiality cannot be guaranteed. Therefore, the researchers have to inform the twins that whilst qualitative data will be anonymised, it will most likely be recognisable to their family members. Furthermore, the participating twins should be informed that they do not need to talk about anything they are uncomfortable talking about or do not want their co-twin to know about, following adequate ethical protocols (see the Belmont report; Adashi et al. 2018).

In conclusion, we conducted a systematic review of qualitative MZ differences literature, involving the application of qualitative data collection and analysis methods to examine differences in experiences of identical twins. Qualitative MZ twin differences design can provide nuance to research on environmental determinants of behaviour by spotlighting lived experiences and distinctiveness, whilst controlling for common systematic sources of variance (i.e., genetics and sociodemographic characteristics) between twins. So far, the use of this study design has been limited and the seven studies that we identified through this review were very heterogeneous. Our narrative synthesis highlighted a range of factors related to twins’ discordance in a range of traits. Some of the factors were differences in personality and individual traits, e.g., confidence or sexual orientation, and in interpersonal relationships. The qualitative MZ differences design is suitable for use in parallel with quantitative twin research in mixed-method research projects or programmes for aims such as inductive exploration prior to quantitative analyses or triangulation of quantitative findings with lived experience insights.

## Supplementary Information

Below is the link to the electronic supplementary material.

Supplementary file1 (DOCX 44 KB)

## Data Availability

No datasets were generated or analysed during the current study.

## References

[CR1] Adashi EY, Walters LB, Menikoff JA (2018) The Belmont report at 40: reckoning with time. Am J Public Health 108(10):1345–1348. 10.2105/AJPH.2018.304580 30138058 10.2105/AJPH.2018.304580 PMC6137767

[CR3] Asbury K, Dunn J, Plomin R (2006) The use of discordant MZ twins to generate hypotheses regarding non-shared environmental influence on anxiety in middle childhood. Soc Dev 15(3):564–570. 10.1111/j.1467-9507.2006.00356.x

[CR4] Asbury K, Moran N, Plomin R (2016) Nonshared environmental influences on academic achievement at age 16: a qualitative hypothesis-generating monozygotic-twin differences study. AERA Open 2(4):2332858416673596. 10.1177/2332858416673596

[CR5] Asbury K, Moran N, Plomin R (2017) Do MZ twins have discordant experiences of friendship? A qualitative hypothesis—generating MZ twin differences study. PLoS ONE. 10.1371/journal.pone.0180521 28727730 10.1371/journal.pone.0180521 PMC5519028

[CR6] Baldwin JR, Wang B, Karwatowska L, Schoeler T, Tsaligopoulou A, Munafò MR, Pingault JB (2023) Childhood maltreatment and mental health problems: a systematic review and meta-analysis of quasi-experimental studies. Am J Psychiatry 180(2):117–126. 10.1176/appi.ajp.20220174 36628513 10.1176/appi.ajp.20220174 PMC7614155

[CR8] Braun V, Clarke V (2006) Using thematic analysis in psychology. Qual Res Psychol 3(2):77–101. 10.1191/1478088706qp063oa

[CR9] Braun V, Clarke V (2023) Is thematic analysis used well in health psychology? A critical review of published research, with recommendations for quality practice and reporting. Health Psychol Rev. 10.1080/17437199.2022.2161594 36656762 10.1080/17437199.2022.2161594

[CR53] Bryman A, Burgess RG (2002) Reflections on qualitative data analysis. In: Analyzing qualitative data. Routledge, pp 216–226

[CR10] Carlin JB, Gurrin LC, Sterne JA, Morley R, Dwyer T (2005) Regression models for twin studies: a critical review. Int J Epidemiol 34(5):1089–1099. 10.1093/ije/dyi153 16087687 10.1093/ije/dyi153

[CR52] Caspi A, Moffitt TE, Morgan J, Rutter M, Taylor A, Arseneault L, Tully L, Jacobs C, Kim-Cohen J, Polo-Tomas M (2004) Maternal expressed emotion predicts children's antisocial behavior problems: using monozygotic-twin differences to identify environmental effects on behavioral development. Dev Psychol 40(2):149. 10.1037/0012-1649.40.2.149 10.1037/0012-1649.40.2.149 14979757

[CR11] Clarke V, Braun V (2017) Thematic analysis. J Posit Psychol 12(3):297–298. 10.1080/17439760.2016.1262613

[CR12] Creswell JW, Clark VP (2011) Mixed methods research. SAGE, Thousand Oaks

[CR13] Critical Appraisal Skills Programme (2013) CASP qualitative studies checklist. [online] Available at: https://casp-uk.net/casp-tools-checklists/ Accessed: 10 Dec 2023

[CR14] Domecka M, Eichsteller M, Karakusheva S, Musella P, Ojamäe L, Perone E et al (2012) Method in practice: autobiographical narrative interviews in search of European phenomena. The evolution of European identities: biographical approaches. Palgrave Macmillan UK, London, pp 21–44

[CR15] Doyle L, Brady AM, Byrne G (2016) An overview of mixed methods research–revisited. J Res Nurs 21(8):623–635. 10.1177/1744987108093962

[CR16] Elwyn R, Williams M, Smith E, Smith S (2024) Two identical twin pairs discordant for longstanding anorexia nervosa and OSFED: lived experience accounts of eating disorder and recovery processes. J Eat Disord 12(1):127. 10.1186/s40337-024-01078-w 39223672 10.1186/s40337-024-01078-w PMC11367789

[CR17] Eriksson H, Harmat L, Theorell T, Ullén F (2017) Similar but different: interviewing monozygotic twins discordant for musical practice. Music Sci 21(3):250–266. 10.1177/1029864916649791

[CR22] Hjelmborg J, Korhonen T, Holst K, Skytthe A, Pukkala E, Kutschke J et al (2017) Lung cancer, genetic predisposition and smoking: the Nordic twin study of cancer. Thorax 72(11):1021–1027. 10.1136/thoraxjnl-2017-211060 29054888 10.1136/thoraxjnl-2015-207921

[CR24] Kendler KS, Halberstadt LJ (2013) The road not taken:life experiences in monozygotic twin pairs discordant for major depression. Mol Psychiatry 18(9):975–984. 10.1038/mp.2012.55 22641178 10.1038/mp.2012.55 PMC3523211

[CR27] Lachal J, Revah-Levy A, Orri M, Moro MR (2017) Metasynthesis: an original method to synthesize qualitative literature in psychiatry. Front Psych 8:269. 10.3389/fpsyt.2017.00269 10.3389/fpsyt.2017.00269 PMC5716974 29249996

[CR28] Levitt HM, Motulsky SL, Wertz FJ, Morrow SL, Ponterotto JG (2017) Recommendations for designing and reviewing qualitative research in psychology: promoting methodological integrity. Qual Psychol 4(1):2. 10.1037/qup0000082

[CR29] Lewis SW, Chitkara B, Revelely AM (1991) Obsessive-compulsive disorder and schizophrenia in three identical twin pairs. Psychol Med 21(1):135–141. 10.1017/S0033291700014720 2047489 10.1017/s0033291700014720

[CR31] McAdams TA, Rijsdijk FV, Zavos HM, Pingault JB (2021) Twins and causal inference: leveraging nature’s experiment. Cold Spring Harb Perspect Med. 10.1101/cshperspect.a039552 32900702 10.1101/cshperspect.a039552 PMC8168524

[CR32] McLeod K, White V, Mullins R, Davey C, Wakefield M, Hill D (2008) How do friends influence smoking uptake? Findings from qualitative interviews with identical twins. J Genet Psychol 169(2):117. 10.3200/GNTP.169.2.117-132 18578296 10.3200/GNTP.169.2.117-132

[CR33] Moriarity J (2011) Methods review 1. School for social care research. http://eprints.lse.ac.uk/41199/1/SSCR_Methods_Review_1-1.pdf

[CR35] Pearsall-Jones JG, Piek JP, Steed L, McDougall MR, Levy F (2011) Monozygotic twins concordant and discordant for DCD: two sides to the story. Twin Res Hum Genet 14(1):79–87. 10.1375/twin.14.1.79 21314259 10.1375/twin.14.1.79

[CR37] Plomin R (2011) Commentary: why are children in the same family so different? Non-shared environment three decades later. Int J Epidemiol 40(3):582–592. 10.1093/ije/dyq144 21807643 10.1093/ije/dyq144 PMC3147062

[CR38] Plomin R, Daniels D (1987) Why are children in the same family so different from one another? Behav Brain Sci 10(1):1–16. 10.1017/S0140525X00055941

[CR54] Ritchie J, Spencer L (2002) Qualitative data analysis for applied policy research. In: Analyzing qualitative data. Routledge, pp 173–194

[CR40] Sağlam M, Çelik OT, Tunç Y, Kahraman Ü, Açar D, Candemir B (2023) Meta-thematic analysis of quality in early childhood education and care. Humanit Soc Sci Commun 10(1):1–12. 10.1057/s41599-023-02491-3

[CR42] Singham T, Viding E, Schoeler T, Arseneault L, Ronald A, Cecil CM et al (2017) Concurrent and longitudinal contribution of exposure to bullying in childhood to mental health: the role of vulnerability and resilience. JAMA Psychiatry 74(11):1112–1119. 10.1001/jamapsychiatry.2017.2678 28979965 10.1001/jamapsychiatry.2017.2678 PMC5710218

[CR44] Sofaer S (1999) Qualitative methods: what are they and why use them? Health Serv Res 34(5 Pt 2):1101–1118 10591275 PMC1089055

[CR45] Terry G, Hayfield N (2021) Essentials of thematic analysis. American Psychological Association, Washington

[CR46] Tikhodeyev ON, Shcherbakova OV (2019) The problem of non-shared environment in behavioral genetics. Behav Genet 49(3):259–269. 10.1007/s10519-019-09950-1 30725340 10.1007/s10519-019-09950-1

[CR47] Treharne GJ, Riggs DW (2015) Ensuring quality in qualitative research. Qual Res Clin Health Psychol 2014:57–73

[CR48] Turkheimer E (2000) Three laws of behavior genetics and what they mean. Curr Dir Psychol Sci 9(5):160–164. 10.1111/1467-8721.00084

[CR49] Turkheimer E, Waldron M (2000) Nonshared environment: a theoretical, methodological, and quantitative review. Psychol Bull 126(1):78. 10.1037/0033-2909.126.1.78 10668351 10.1037/0033-2909.126.1.78

[CR51] Yardley L (2017) Demonstrating the validity of qualitative research. J Posit Psychol 12(3):295–296. 10.1080/17439760.2016.1262624

