# Supplementary material for: Towards a Greater Understanding of the Role of the Environment: A Systematic Review of Qualitative MZ Twin Differences Studies
Source: Behav Genet. 2025 Feb 23;55(3):153–68. doi: 10.1007/s10519-025-10217-1 (PMC12043727; doi:10.1007/s10519-025-10217-1)
Supplement: Supplementary file 1 — Supplementary file1 (DOCX 44 KB) [file 10519_2025_10217_MOESM1_ESM.docx]

**Article title:** Investigating Environmental Influences: A Systematic Review of Qualitative MZ Twin Differences Studies and Recommendations for Future Studies

**Journal:** Behavior Genetics

**Author Names**: Filip Marzecki ^1,2^ *, Kennath Widanaralalage ^1^, Nandini Bhandoh ^1^, Tom A. McAdams ^2^, Yasmin I. Ahmadzadeh ^2^, Helena M. S. Zavos ^1^

**Author Affiliations:** ^1^ Department of Psychology, Institute of Psychiatry, Psychology & Neuroscience, King’s College London, London, UK. ^2^ Social, Genetic & Developmental Psychiatry Centre, Institute of Psychiatry, Psychology & Neuroscience, King’s College London, London, UK. *Corresponding author

**Correspondence:** Mr Filip Marzecki, email: [filip.kaleta@kcl.ac.uk](mailto:filip.kaleta@kcl.ac.uk)

**Supplementary Materials**

**Appendix 1**. Search Strategy

OVID (APA PsycINFO, 1806 - 2023):

Concept 1 – Population:

MZ twin* OR identical twin* OR monozygotic twin* OR monozygous twin* OR Co-twin control OR co-twin method

AND

Concept 2 - Research Method:

qualitative study OR qualitative research OR qualitative method* OR Interview* OR focus group*

AND

Concept 3 - Design:

Different OR difference* OR Discordant OR discordance*

PubMed (1998-2023):

Concept 1 – Population:

MZ twin* OR identical twin* OR monozygotic twin* OR monozygous twin* OR Co-twin control OR co-twin method

AND

Concept 2 - Research Method:

qualitative study OR qualitative research OR qualitative method* OR Interview* OR focus group*

AND

Concept 3 - Design:

Different OR difference* OR Discordant OR discordance*

**Appendix 2**. Glossary

**Thematic Analysis** – a family of common qualitative data analysis methods, which emphasise identifying, analysing and interpreting patterns of meaning

**Framework Approach** – a type of thematic analysis (also called codebook thematic analysis), which involves coding the data according to a pre-determined codebook

**Methodology** - the theory of how research needs to be conducted to lead to valid knowledge, i.e., it provides a framework for decisions about participant selection, data collection, and interpretation of the findings. Choosing a methodology is driven by philosophical stances, such as on epistemology (the “theory of knowledge”).

**Interpretative Phenomenological Analysis** - a qualitative method and methodology, suitable when researchers are interested in illustrating how people make sense of their experiences; rooted in the epistemological perspective of social constructivism.

**Epistemological Stance** – a position regarding epistemology: the “theory of knowledge” which concerns the distinctions between truth, belief and opinion.

**Positivism** – an epistemological stance that considers truth to exist objectively and independently of other factors, such as social systems.

**Social Constructivism** – an epistemological stance that considered knowledge to be constructed by people’s interactions with others

**Transferability** - considering whether and what other contexts are the findings applicable in

**Positionality** - the researcher clearly stating their position, e.g., their beliefs, about the research topic, design, and the participants

**Reflexivity** - the examination of the beliefs and judgments of the researcher and how these may influence the research

**Supplementary Table 1**. Themes or Categories of Data in Included Studies

| Author and Date | Discordant trait | Themes |
| --- | --- | --- |
| Asbury, Moran and Plomin, 2016 | Academic achievement at 16 | Two themes: **School environment** (Ability grouping, Teacher quality, Teacher–pupil relationships), **Individual traits/behavior** (Ability, Personality, Effort and motivation, Interest or enjoyment) |
| Asbury, Moran and Plomin, 2017 | Peer relationships | Analysis of NSE influence on discordance resulted in different themes depending on the type of discordance. The NSE influence on **discordance in peer victimiation and rejection** were themed as **chance** and one twin's **enhanced vulnerability**. The themes of NSE influences on **discordance in the number of friends** were **personality** (including confidence and self-esteem), **health-related enhanced vulnerability** (leading to absences from school), **chance** and **romantic relationships**. The themes of NSE influences on discordance in friendship groups were **chance, choice, personality** (including confidence and interests) and parental encouragement. The themes of NSE influences on **discordance in different attitudes to friendship** were **discordant efforts to socialise, personality** (need for approval, confidence), and **reactions to the twin relationship**. |
| Asbury, Dunn and Plomin, 2006 | Anxiety | Six themes of NSE sources of discordance were identified: **school experiences, comparisons within the twin relationship, and illnesses and accidents, traumatic neonatal events** (breathing problems, separation from mother), **parent-child relationships**, and **peer rejection**. |
| Eriksson, Harmat, Theorell and Ullen, 2016 | Keyboard instrument playing | The authors did not formally build themes from the data. The categories of NSE influences on musical engagement were differences in access to a piano, either while growing up or in adult life; differences in attitudes towards the musical genres performed; differences in the perceived need for a creative hobby; differences in social activities and musical interests among friends; different feelings about the common music teacher; and different attitudes towards music as an expression of religious faith. These could be further grouped as **access to a piano, personality**, and **social interactions** (friendship groups and music teachers). |
| Kendler and Halberstadt, 2013 | Major Depressive Disorder (MDD) | Themes of NSE influences on discordance were **romantic relationships, being planful**, as well as **occupational stressors** (e.g., lack of a stable career) and **single traumatic events**. |
| McLeod, White, Mullins, Davey, Wakefield and Hill, 2008 | Smoking | The authors did not share formal themes developed from their data, but NSE influences investigated here were pertaining specifically to social experiences, therefore **social factors** (e.g., peer pressure) as well as **personality and identity differences** in relating to social groups and images were brought up. |
| Pearsall-Jones, Piek, Steed, McDougall and Levy, 2011 | Developmental Coordination Disorder (DCD) | The effects of discordance in DCD on perceived family dynamics, and especially the twin relationship, were grouped into the following themes: **friendship, support, minimal sibling rivalry**, and **minor difficulties**. |
